# Endothelium-derived microparticles from chronically thromboembolic pulmonary hypertensive patients facilitate endothelial angiogenesis

**DOI:** 10.1186/s12929-016-0224-9

**Published:** 2016-01-19

**Authors:** Daria Belik, Hilda Tsang, John Wharton, Luke Howard, Carmelo Bernabeu, Beata Wojciak-Stothard

**Affiliations:** Centre for Pharmacology and Therapeutics, Department of Medicine, Imperial College London, London, UK; National Pulmonary Hypertension Service, Imperial College Healthcare NHS Trust, London, UK; Centro de Investigaciones Biológicas, Consejo Superior de Investigaciones Científicas (CSIC), and Centro de Investigación Biomédica en Red de Enfermedades Raras (CIBERER), 28040 Madrid, Spain

**Keywords:** Endoglin, Angiogenesis, Pulmonary hypertension, Microparticles

## Abstract

**Background:**

Increased circulating levels of endoglin^+^ endothelial microparticles (EMPs) have been identified in several cardiovascular disorders, related to severity. Endoglin is an auxilary receptor for transforming growth factor β (TGF-β) important in the regulation of vascular structure.

**Results:**

We quantified the number of microparticles in plasma of six patients with chronic thromboembolic pulmonary hypertension (CTEPH) and age- and sex-matched pulmonary embolic (PE) and healthy controls and investigated the role of microparticle endoglin in the regulation of pulmonary endothelial function *in vitro*. Results show significantly increased levels of endoglin^+^ EMPs in CTEPH plasma, compared to healthy and disease controls. Co-culture of human pulmonary endothelial cells with CTEPH microparticles increased intracellular levels of endoglin and enhanced TGF-β-induced angiogenesis and Smad1,5,8 phosphorylation in cells, without affecting BMPRII expression. In an *in vitro* model, we generated endothelium-derived MPs with enforced membrane localization of endoglin. Co-culture of these MPs with endothelial cells increased cellular endoglin content, improved cell survival and stimulated angiogenesis in a manner similar to the effects induced by overexpressed protein.

**Conclusions:**

Increased generation of endoglin^+^ EMPs in CTEPH is likely to represent a protective mechanism supporting endothelial cell survival and angiogenesis, set to counteract the effects of vascular occlusion and endothelial damage.

**Electronic supplementary material:**

The online version of this article (doi:10.1186/s12929-016-0224-9) contains supplementary material, which is available to authorized users.

## Background

Chronic thromboembolic pulmonary hypertension (CTEPH) is one of the leading causes of severe pulmonary hypertension (PH). In CTEPH, the formation of secondary nonresolving thromboemboli following the acute phase of thrombotic pulmonary embolism, leads to the obstruction of the pulmonary vascular bed followed by vascular remodelling and right heart hypertrophy [1, 2]. Endothelial dysfunction and defective thrombus neovascularization accompanied by a decrease in the expression of factors involved in proliferative pathways of vascular cells, such as bone morphogenetic protein receptor type 2 (BMPR2) or TGF-β1, are thought to play a key role in the pathogenesis of CTEPH [3].

Circulating plasma microparticles (MPs) have been implicated in the pathogenesis of numerous cardiovascular disorders including pulmonary arterial hypertension (PAH), but their cellular origin and associated specific roles have not been fully elucidated [4]. MPs are <1 μm membrane vesicles released after cell activation or apoptosis [5–7]. Depending on the size, formation and release mechanism, microparticles can be divided into 2 groups: exosomes and microvesicles. Exosomes (40-100 nm in diameter) derive from multivesicular bodies, which are compartments of the endosomal system, while microvesicles (100-1000 nm in diameter) derive from plasma membrane via shedding [8]. MPs harbor membrane proteins of the parent cells and contain intracellular signalling molecules such as microRNA or DNA fragments [7, 9].

Increased levels of endoglin^+^ (CD105^+^) endothelium-derived MPs (EMPs) can be detected in blood from remodelled pulmonary arterial hypertensive (PAH) lung [10] and occluded coronary arteries [11, 12]. Endoglin is an ancillary receptor for several TGF-β superfamily ligands, including bone morphogenetic proteins (BMPs) [13]. Defective signaling of TGF-β family of proteins is common to most forms of PH [14], including CTEPH [2]. Vascular injury, inflammation and hypoxia, in particular in combination with TGF-β are strong inducers of endoglin expression [13]. Ectopic expression of endoglin promotes endothelial cell proliferation and inhibits hypoxia-induced endothelial cell apoptosis via TGF-β/Alk-1 signaling [15, 16]. Inhibition of endoglin signalling is associated with pulmonary vascular remodelling in pulmonary hypertension. Adult *Eng*^*+/−*^ mice spontaneously develop signs of pulmonary hypertension that are attributable to uncoupled eNOS activity and reactive oxygen species (ROS) production causing progressive loss in pulmonary vascularity and increased muscularization of arterioles [17]. Interestingly, circulating levels of a soluble, truncated form of endoglin (Sol.Eng) are elevated in PAH and preeclampsia, hypercholesterolemia, atherosclerosis and acute myocardial infarction [17–19]. It has been postulated that Sol.Eng inhibits TGF-βR2-dependent signalling by binding circulating TGF-β and attenuating vasorelaxation [20].

We hypothesized that CTEPH MPs can alter the function of healthy human pulmonary endothelial cells leading to changes that may affect disease progression. To address this hypothesis, we compared the effects of MPs isolated from plasma of CTEPH patients, pulmonary embolic patients and healthy individuals, on pulmonary endothelial survival, proliferation and angiogenesis *in vitro*. The effect of microparticle endoglin on endothelial function was studied with the use of microparticles from plasma and endothelial cells overexpressing recombinant endoglin.

## Methods

### Blood collection and patient information

Venous blood samples were obtained with local ethics committee approval and informed written consent from 6 CTEPH patients, 6 pulmonary embolic (PE) patients and 4 healthy individuals with no history of PE or PH (Table 1).

**Table 1:** Patient characteristics

	Healthy	CTEPH	PE
	*N* = 4	*N* = 6	*N* = 6
Males/females	2/2	2/4	2/4
Age, years	54 (42–60)	68 (52–81)	68 (45–91)
mPAP, mmHg	-	42 (30–57)	-
Warfarin/anticoagulants	-	6	6
Ca channel blockers	-	1	1
statins	-	2	2
paracetamol/aspirin	-	3	4
ET-1 antagonists	-	5	-
Diuretics	-	4	1
ACE inhibitors	-	2	1
Angiotensin II antagonists	-	-	1
β-blockers	-	1	4

### Isolation and characterization of microparticle fraction from human plasma

25 mL of systemic venous blood was collected in the presence of 3.8 % sodium citrate and subjected to sequential centrifugation at 1,500 g for 15 min, 5,000 g for 5 min, 10,000 g for 30 min and 200,000 g for 60 min. PKH26 Red Fluorescent Cell Linker Kit (Cat# MINI26 and PKH26GL, Sigma, USA) was used to label the MPs according to the manufacturer’s protocol. In order to prepare MPs for the flow cytometry analysis, the mixtures were diluted with filtered PBS (1:3) and 3 μm latex polystyrene beads (Ref# LB30-1ML, Sigma) were added. Phycoerythrin-conjugated anti-human VE-cadherin (CD144, cat. no c-1449-80, eBioscience, USA) was used to label endothelium- derived MPs. Human Annexin 5-FITC antibody (cat. no BMS306FI/20, eBioscience) was also used to label the phosphatidylserine-rich MPs.

### Scanning electron microscopy

Human pulmonary artery endothelial cells (HPAECs) were plated on coverslips and incubated with purified MP fraction for 30 min. The cells were then fixed in 4 % formaldehyde in PBS for 15 min, washed with PBS and dehydrated by subsequent incubation in 20 %, 50 %, 70 %, 80 %, 90 % and 100 % ethanol; 5 min in each solution. The cells were transferred into hexamethyldisilazane (HMDS) for 5 min and left to air dry. The samples were gold-coated for one minute at 20 mA and analysed with laser scanning microscope JEOL JSM-5610LV (JEOL, Japan) at 15 KV in secondary imaging mode (SEI) and magnified images were taken with Helios 600 NanoLab (FEI Electron Microscopes, USA).

### Quantification of microparticles by flow cytometry

Fluorescently labelled MPs were analysed with FACSCalibur flow cytometer (BD Biosciences, USA). MP concentration in plasma was calculated from the equation:

$$ \begin{array}{c}\hfill \mathrm{Estimated}\hfill \\ {}\hfill \mathrm{M}\mathrm{P}/\mathrm{mL}\kern0.5em \mathrm{of}\kern0.5em \mathrm{plasma}\hfill \end{array}=\frac{(203111)/\left(\mathrm{no}.\kern0.5em \mathrm{of}\kern0.5em \mathrm{beads}\kern0.5em \mathrm{counted}\right)*\left(\mathrm{no}.\kern0.5em \mathrm{of}\kern0.5em \mathrm{M}\mathrm{P}\kern0.5em \mathrm{counted}\right)*\mathrm{x}}{\mathrm{Volume}\kern0.5em \mathrm{of}\kern0.5em \mathrm{plasma}\kern0.5em \mathrm{used}\kern0.5em \left(\mathrm{mL}\right)} $$

3 μm polystyrene beads were used to estimate the concentration of MPs per mL of plasma. 203111 is a constant, provided by the manufacturer. The number of beads counted is the number of events identified as beads due to their size and characteristic density plot. Number of MP counted is the number of events detected as MPs due to positive staining. X is the dilution factor.

### Quantification of microparticles by laser light scattering spectroscopy

0.3 mL of the MP suspensions were analysed with the Nanosight LM10-HSGFT14 Nanoparticle Analysis System (Nanosight, UK), using Nanoparticle Tracking Analysis (NTA) software (Nanosight).

### Quantification of endoglin in microparticle fractions

Endoglin was quantified in MP fractions using Human Endoglin/CD105 Quantikine ELISA Kit (R&D), according to the manufacturer’s protocol.

### Cell culture

HPAECs (PromoCell GmbH, Germany) were cultured in endothelial cell growth medium 2 (ECGM2) (PromoCell GmbH, Germany) containing 2 % foetal calf serum, growth factor supplement mix and penicillin/streptomycin in tissue culture dishes coated with 10 μg/mL bovine fibronectin (Sigma) in a humidified incubator (5 % CO_2_) at 37 °C.

### Endoglin expression vectors

The pDisplay-HA-L-endoglin expression vector containing the HA-tagged human full length (L)-endoglin has been described in [21]. pDisplay™ (Invitrogen, USA) is a 5.3 kb mammalian expression vector that allows display of proteins on the cell surface. Proteins expressed from pDisplay™ are fused at the N-terminus to the murine Ig κ-chain leader sequence, which directs the protein to the secretory pathway. The pCEXV-HA-L-endoglin expression vector contains the HA-tagged full length human L-endoglin, including its leader sequence and will be described elsewhere. Empty pCEEXV vector and empty pDisplay vector were used as transfection controls. HPAECs were transfected using Amaxa™ Basic Nucleofector™ Kit for Primary Mammalian Endothelial Cells (Lonza, Switzerland) applying optimal Nucleofector™ program M-003. The cells were used for experiments 18 h post-transfection.

### *In vitro* model of MP generation

MPs were generated by an established method of serum starvation [22]. The untransfected or transfected HPAECs were grown in T75 flasks in 10 mL of serum- and growth factor-depleted medium. Following 24 h incubation, MPs were isolated from HPAEC conditioned media by sequential centrifugation.

### HPAEC-MP co-culture experiments

In experiments involving co-culture of HPAECs with MPs, MPs obtained from 2 mL plasma were re-suspended in 2 mL of culture medium and plated on cells. Endoglin^−^ or VE-cadherin^−^ MP fractions were obtained by antibody-mediated immunoprecipitation with Dynabeads (Life Technologies). Briefly, 2 mL of MP suspension was incubated o/n with 30 μL Dynabeads linked with 5 μg of mouse anti-human endoglin (Millipore, USA) or mouse monoclonal anti-VE-cadherin antibody (Santa Cruz Biotechnology). The suspension was then placed in a magnetic holder and the supernatant was carefully collected without disturbing Dynabeads attached to the side of the tube. The beads were washed 3x in PBS. All samples (the beads, full MP fraction, VE-cadherin^−^ and endoglin^−^ MPs) were resolved by SDS-PAGE (30 μg protein/lane) and studied by western blotting. Endoglin levels were also measured in ELISA assay, as described above.

In co-culture experiments, HPAECs grown in 96-well plates were incubated with MP suspension obtained from patient plasma or from endoglin-overexpressing HPAECs. In some experiments, TGF-β (10 ng/mL) was added to the cells 1 h after the addition of MPs and incubated for further 1 h (smad phosphorylation) or 18 h (cell proliferation, angiogenesis and BMPRII expression).

### Immunostaining and confocal laser scanning microscope

HPAECs grown on plastic coverslips in 24-well plates were incubated with MPs for 1–18 h, as appropriate. The cells were then fixed in 4 % formaldehyde in PBS, permeabilised with 0.1 % Triton X-100 and incubated with 2 % bovine serum albumin (BSA) to block non-specific antibody binding [23]. The coverslips were incubated with 50 μL of mouse anti-endoglin monoclonal antibody (10 μg/mL, Millipore, USA) and rabbit anti-HA polyclonal antibody (5 μg/mL, Santa Cruz Biotechnology, USA) in PBS for 1 h. Coverslips were then washed in PBS and incubated with TRITC (Tetramethylrhodamine-5-(and 6)-isothiocyanate)-phalloidin (1:100, Sigma) and Cy5-conjugated goat anti-mouse and FITC (Fluorescein isothiocyanate)-conjugated goat anti-rabbit antibody (10 μg/mL, Invitrogen, USA) for 1 h, washed in PBS and mounted in Vectashield mountant with nuclear stain DAPI (Vector Laboratories, USA). Fluorescent confocal images were taken using Leica TCS SP5 (Leica Microsystems, Germany).

### Western blotting

Following electrophoresis and protein transfer, PVDF membranes were incubated overnight with mouse monoclonal anti-endoglin antibody (1 μg/mL, R&D, USA), mouse monoclonal anti-HA-probe antibody (1 μg/mL, Santa Cruz Biotechnology, USA), mouse monoclonal anti-VE-cadherin antibody (1 μg/mL, Santa Cruz Biotechnology, USA), mouse monoclonal anti-BMPRII antibody (0.5 μg/mL, R&D), mouse monoclonal anti- β-actin antibody (0.2 μg/mL, Sigma), rabbit anti-p-smad1,5,8 (0.5 μg/mL; Santa Cruz Biotechnology, USA) or mouse monoclonal anti-smad 1 antibody (0.5 μg/mL; Santa Cruz Biotechnology, USA). The membranes were washed in Tris-buffered saline with Tween 20, incubated with goat anti-mouse- or goat anti-rabbit polyclonal HRP-conjugated antibodies (0.2 μg/mL; DAKO, USA) for 1 h. Blots were developed in Luminata Crescendo Western HRP substrate (Millipore, USA) and analysed in BioRad ChemiDoc Imager.

### Angiogenesis assay

Untransfected or transfected HPAECs were seeded on 40 μL of growth factor-reduced Matrigel (Corning™, # 354230) in a 96-well plate (14 × 10^3^ cells per well) in growth factor-free medium containing 0.5 % FBS, with or without MPs (full fraction, VE-cadherin^−^ or endoglin^−^) or TGF-β (10 ng/mL), as appropriate. Following 18 h incubation, the cells were fixed in 4 % formaldehyde in PBS and photographed under a phase contrast microscope (Olympus IX70, Japan) equipped with a Peltier CCD camera. Total tube length was calculated using Image J software.

### Proliferation assay (MTS)

HPAECs (7 × 10^3^ cells per well) were cultured in 96-well plates in growth factor-free medium containing 0.5 % FBS, with or without MPs and TGF-β (10 ng/mL), as appropriate. Following 18 h incubation, cell proliferation was measured using CellTiter 96® Aqueous One Solution Cell Proliferation Assay System (Promega, USA). In this assay, the conversion of MTS tetrazolium compound into a coloured formazan product is accomplished by NADPH or NADH generated by dehydrogenase enzymes in metabolically active cells. The quantity of the coloured formazan product determined by absorbance at 490 nm is directly proportional to the number of living cells and commonly associated with cell proliferation and migration [24].

### Apoptosis assay

Untransfected or transfected HPAECs were cultured overnight in 96-well optical bottom plates at cell density of 7 × 10^3^ cells per well in growth factor-free medium containing 0.1 % foetal calf serum, with or without MPs, as appropriate. 100 μL of DiOC7(3) (3,3′-Diheptyloxacarbocyanine Iodide), a cell-permanent, green fluorescent, lipophilic dye that selectively labels the mitochondria in live cells (1: 1000 dilution in PBS, Life Technologies, Invitrogen, USA) was added into each well. After 45 min incubation at 37 °C, fluorescence was read at excitation/emission 490/525 nm in GloMax®-Multi + Microplate Multimode Reader (Promega, USA).

### Human angiogenesis microarray

The levels of pro-angiogenic cytokines in CTEPH EMP fraction were studied with Proteome Profiler™ Human Angiogenesis Array (R&D Systems™, # ARY007), according to the manufacturer’s protocol. Briefly, EMPs were removed from 5 × 10^6^ CTEPH MPs fraction by Dynabeads antibody capture, as described above. Following 3 washes in PBS, Dynabeads with the bound EMPs were re-suspended in 1 ml of PBS and incubated overnight with the human angiogenesis microarray membrane. More information about the assay can be found on https://resources.rndsystems.com/pdfs/datasheets/ary007.pdf.

### Ethics, consent and permissions

All procedures performed in studies involving human participants were in accordance with the ethical standards of the institutional and/or national research committee and with the 1964 Helsinki declaration and its later amendments or comparable ethical standards.

Informed consent was obtained from all individual participants included in the study.

### Statistical analysis

All experiments were performed in triplicate or, as indicated. Data was analysed using one-way ANOVA (GraphPad Prism Version 6) followed by Tukey’s post-test.

## Results

### CTEPH patients show increased levels of endoglin^+^ endothelium-derived microparticles, compared with healthy and disease controls

**Fig. 1:**
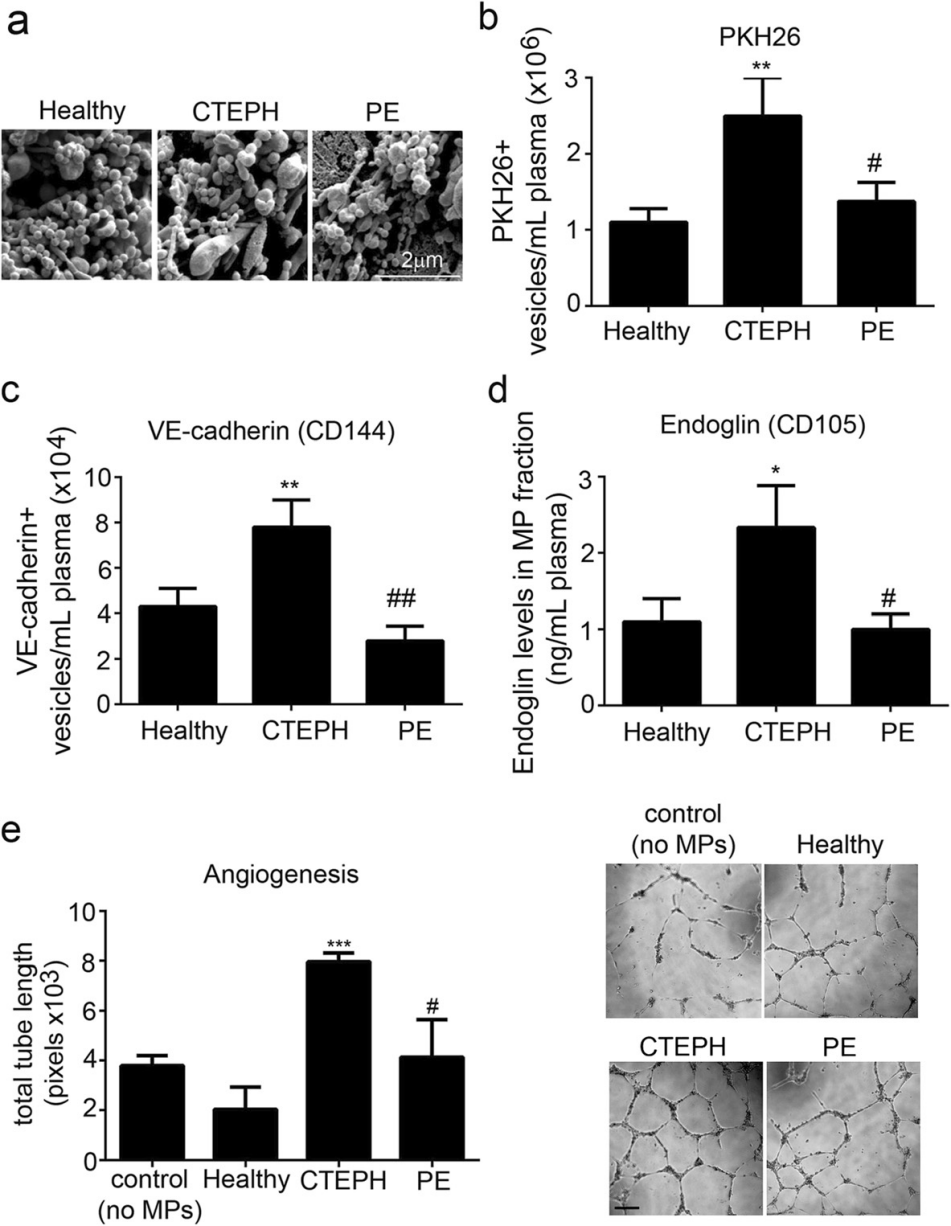
CTEPH plasma shows increased levels of endothelium-derived microparticles compared with pulmonary embolic and healthy controls. **a** Scanning electron microscope images of MP fractions.from a healthy volunteer, CTEPH and PE patients. Bar = 2 μm. **b** Number of membrane (PKH26^+^) MPs and **c** number of VE-cadherin^+^ (CD144^+^) MPs in plasma of healthy voulnteers, CTEPH and PE patients, as indicated; **d** Endoglin levels in MP fractions; ELISA assay; **e** endothelial angiogenesis (Matrigel tube formation) in HPAECs cultured with MPs isolated from CTEPH, PE or healthy plasma, as indicated. **P < 0.01; *P < 0.05, comparisons with healthy controls, ^##^ *P* < 0.01; ^#^ *P* < 0.05, comparisons with CTEPH; *N* = 4-6. Representative images of tube formation are shown beside the graph. Bar = 50 μm

Electron scanning microscopy analysis confirmed vesicular appearance and sizes ranging from 0.01 to 1.5 μm of MPs purified from patient plasma (Fig. 1a). Flow cytometry analysis revealed significantly higher number of annexin V^+^ PKH26^+^ MPs in CTEPH plasma compared to pulmonary embolic patients and healthy controls (2.5 × 10^6^ MPs/mL compared to 1.4 × 10^6^ MPs/mL and 1.1 × 10^6^ MPs/mL respectively) (Fig. 1b). The number of endothelial (VE-cadherin^+^) MPs (EMPs) in CTEPH plasma was 7.5 × 10^4^ EMPs/ml (~3 % of total MP fraction), which was ~2-2.8-fold higher than the number of EMPs found in control groups (Fig. 1c). MP fraction from CTEPH patients also contained 2.5-fold higher levels of endoglin, compared with healthy and PE controls (Fig. 1d). While the levels of endoglin in CTEPH microparticle fraction were elevated, there were no significant differences in the total plasma levels of endoglin between the studied groups (4.1 ± 0.5 ng/mL in healthy plasma, 3.9 ± 0.4 ng/mL for CTEPH and 3.2 ± 0.5 ng/mL for PE).

### CTEPH MPs stimulate endothelial angiogenesis *in vitro*

We further sought to establish the effect of MPs on human pulmonary endothelial function *in vitro*. MPs were added to HPAEC cultures at the concentration found in plasma and incubated for 1-18 h. Confocal microscopy analysis confirmed that microparticles were internalised by the cells within the first hour of incubation (data not shown).

Co-culture of HPAECs with CTEPH microparticles increased endothelial angiogenesis (tube formation) by ~2.5-fold (*P* < 0.001, comparison with healthy and disease controls) and the effect was dose-dependent (Additional file 1: Figure S1).

### Endoglin^+^ endothelium-derived MPs (EMPs) from CTEPH blood enhance TGF-β-induced angiogenesis and Smad1,5,8 phosphorylation in HPAECs

**Fig. 2:**
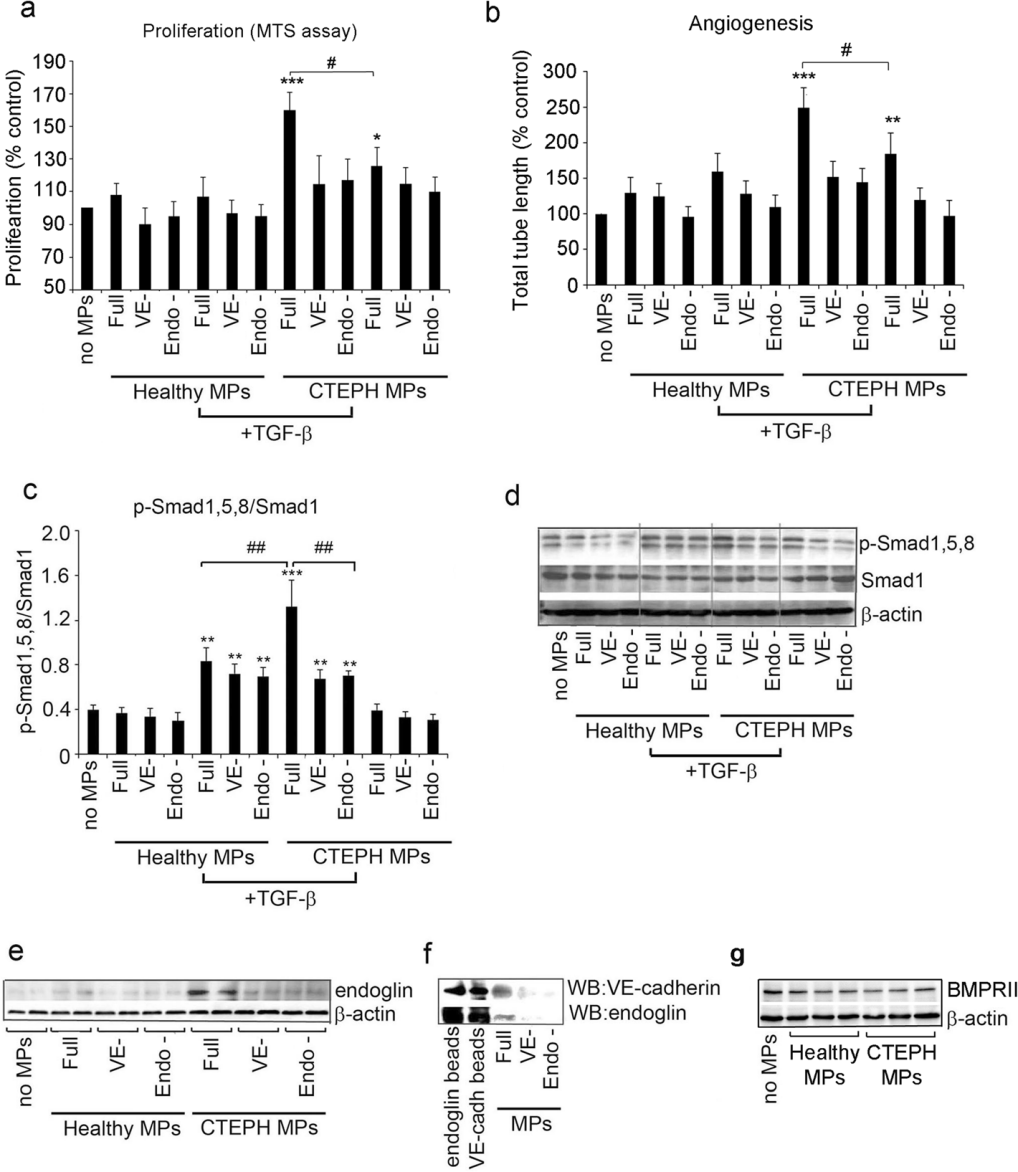
Endoglin^+^ EMPs increase cell proliferation, angiogenesis and TGF-β signalling in HPAECs. **a** Proliferation and (**b**) angiogenesis in HPAECs incubated with unmodified (full), VE cadherin- depleted (VE^−^) or endoglin- depleted (endoglin^−^) MP fractions, in the absence or in the presence of TGF-β (10 ng/mL; 18 h), as indicated. Graph in (**c**) and a corresponding, representative western blot in (d) show phosphorylation changes of smad 1/5/8 in cells pre-incubated with MPs for 1 h and then treated with 10 ng/mL TGF-β for 1 h. *P < 0.05; **P < 0.01; ***P < 0.001, comparisons with healthy controls; ^#^ *P* < 0.05, ^##^ *P* < 0.01 comparisons, as indicated. *N* = 3. **e** Changes in endoglin expression in HPAECs cultured for 18 h with unmodified (full), VE^−^ and endoglin^−^ MPs from healthy volunteers or CTEPH patients. **f** BMPRII expression in HPAECs incubated with MPs for 18 h. **g** Western blot showing VE-cadherin (CD144) and endoglin (CD105) levels in MP fractions, as indicated. Briefly, plasma MPs were incubated o/n with Dynabeads coupled with antibodies against CD144 or CD105. The beads with captured proteins were collected and washed in PBS. Then the beads and, full MP fraction and endoglin/VE-cadherin-depleted fractions were resolved by electrophoresis. Changes in endoglin and VE-cadherin levels were studied by western blotting

CTEPH MPs stimulated proliferation and tube formation in the untreated and TGF-β-treated HPAECs (10 ng/mLTGF-β, 18 h incubation). The stimulatory responses were attenuated by removing endoglin^+^ and VE cadherin^+^ MPs from the total MP fraction (Fig. 2a, b and Additional file 1: Figure S2). CTEPH MPs significantly increased TGF-β-induced Smad1/5/8 phosphorylation in HPAECs, likely to indicate enhanced endoglin signalling (Fig. 2c, d). This response was prevented by removing endoglin^+^ VE-cadherin^+^ MPs from the total MP fraction (Fig. 2d).

### Microparticle-mediated transfer of membrane endoglin facilitates the pro-angiogenic and anti-apoptotic effects of MPs

Incubation of HPAECs with the full (unmodified) MP fraction from CTEPH plasma increased endoglin expression in HPAECs, while endoglin- and VE-cadherin-depleted fractions had no significant effect (Fig. 2e). Depleting microparticles of VE-cadherin removed endoglin and *vice versa*, depleting microparticles of endoglin removed VE-cadherin from MP fraction (Fig. 2f and Additional file 1: Figure S3), indicating that the endothelium-derived (VE-cadherin^+^) MPs were the main carriers of endoglin. Preliminary microarray analysis of CTEPH EMP fraction (*n* = 2) showed the presence of plasminogen activator inhibitor 1 (Serpine1) and traces of urokinase plasminogen activator (uPA) but did not detect measurable levels of any other pro-angiogenic factors included in the assay (Additional file 1: Figure S4).

To verify whether the membrane-bound endoglin plays a role in MP-induced responses, we overexpressed L-endoglin in HPAECs using the mammalian expression vector pDisplay that allows display of this protein on the cell surface [21]. pDisplay-HA-L-endoglin vector contains the HA-tagged full length human L-endoglin with the leader sequence of Igk-chain. To investigate potential role of the membrane localization of L-endoglin, we also used another expression vector, pCEXV-HA-L-endoglin that encodes the own leader sequence of endoglin. Empty vectors were used as transfection controls.

**Fig. 3:**
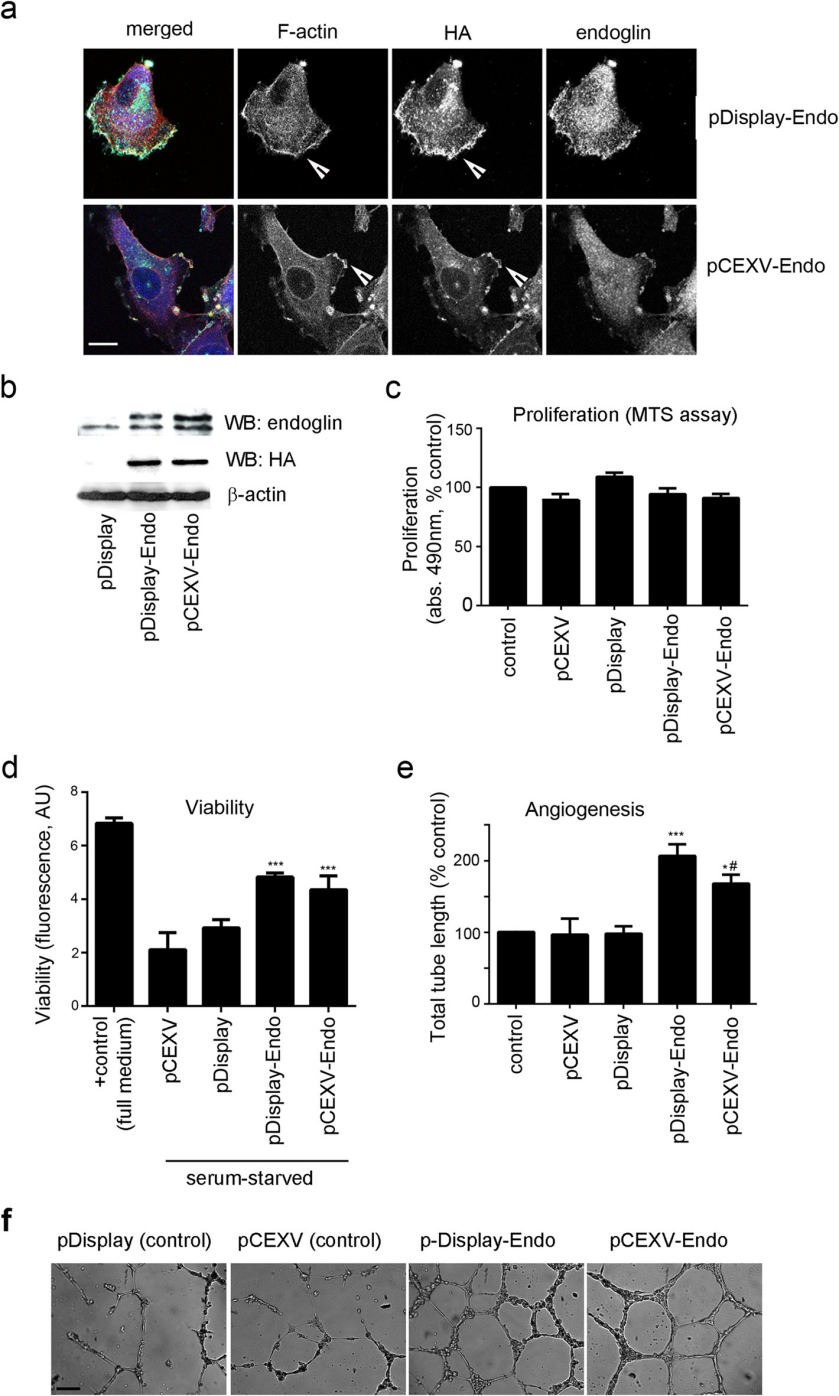
L-endoglin induces pro-survival and pro-angiogenic responses in HPAECs. **a** Cellular localization of endoglin and F-actin in HPAECs transfected with pDisplay-HA-L-endoglin and pCEXV-HA-L-endoglin following an overnight incubation. The arrows in (**a**) point to the lamellipodia where recombinant endoglin co-localises with F-actin. In the merged image, F-actin is red, endoglin is green and HA-tag is blue. Bar = 10 μm. **b** Corresponding western blot shows expression levels of HA-tagged endoglin, total endoglin and β-actin in HPAECs transfected with empty pDisplay, pDisplay-HA-L-endoglin and pCEXV-HA-L-endoglin, as indicated. **c** Cell proliferation; (**d**) cell viability and (**e**) angiogenesis (tube formation) in HPAECs overexpressing pDisplay-HA-L-endoglin and pCEXV-HA-L-endoglin or transfected with control empty vectors, as indicated. In (**d**) cells were serum-starved (0.1 % serum; 18 h), while the non-starved (incubated with full culture media) cells served as a positive (+) control. In (**e**) HPAECs were cultured in growth factor-depleted and serum-reduced (0.5 % serum) media. *P < 0.05; ***P < 0.001, comparisons with vector controls; #*P* < 0.05, comparison with pDisplay-L-endoglin. *N* = 4. **f** Representative images of tube formation in cells treated, as indicated. Bar = 50 μm

Recombinant endoglin localised in F-actin-rich membrane protrusions along the leading edge of migrating endothelial cells (Fig. 3a), similar to the localization observed in human prostate cancer cells [25]. Transfection of cells with pDisplay-HA-L-endoglin and pCEXV-HA-L-endoglin (~70 % transfection efficiency) increased endoglin levels by ~2-fold (Fig. 3b). Overexpression of pDisplay-HA-L-endoglin or pCEXV-HA-L-endoglin did not affect cell proliferation but stimulated tube formation and inhibited starvation-induced apoptosis in HPAECs (Fig. 3c-f), with pDisplay-HA-L-endoglin having a more pronounced effect.

**Fig. 4:**
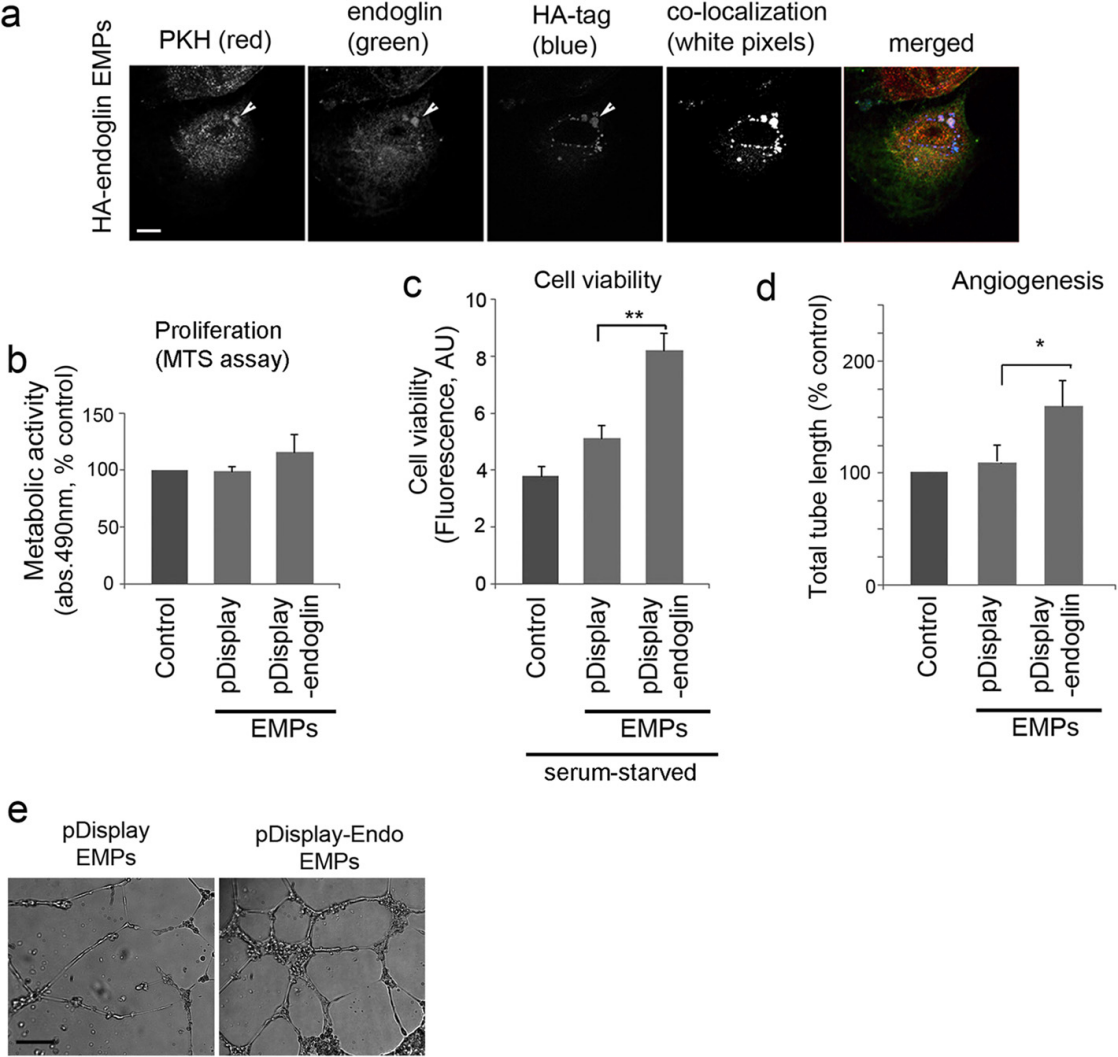
MPs obtained from endoglin-overexpressing HPAECs stimulate endothelial cell survival and angiogenesis *in vitro*. **a** Internalisation of fluorescently-labelled MPs by HPAECs (2 h incubation). In the merged image, PKH26 is red, HA is blue and endoglin is green, as indicated. White pixels mark co-localization of PKH26, HA and endoglin and indicate intracellular localization of microparticles carrying recombinant endoglin (Image J analysis). Bar = 2 μm. **b** Cell proliferation, (**c**) cell viability and (**d**) angiogenesis/tube formation in HPAECs treated with MPs isolated from untreated (control) HPAECs, cells transfected with empty pDisplay vector and HPAECs overexpressing pDisplay-L- endoglin, as indicated. **e** Representative images of tube formation in cells transfected with pDisplay or pDisplay-L-endoglin.*P < 0.05; ***P < 0.001, comparison with transfection controls; Bar = 50 μm; *n* = 3

We further aimed to explore whether endoglin effects can be passed onto cultured cells by MPs generated from endoglin-overexpressing HPAECs. To generate MPs, L-endoglin overexpressing HPAECs were starved for 24 h in serum- and growth factor-free media. The cells produced an average of 0.2-0.32 × 10^6^ MPs/mL of medium. These microparticles were then incubated with HPAECs in a similar manner to plasma-derived MPs. Confocal microscopy analysis documented a transfer of HA-endoglin-bearing MPs to the recipient cells (Fig. 4a). Incubation of HPAECs with microparticles generated by pDisplay-HA-L-endoglin-overexpressing cells inhibited starvation-induced apoptosis and significantly increased endothelial tube formation in HPAECs, while control MPs (generated by HPAECs transfected with an empty pDisplay vector) did not have a significant effect (Fig. 4b-e).

## Discussion

We are first to demonstrate that CTEPH patients show increased levels of circulating endoglin^+^ EMPs compared with healthy and pulmonary embolic control groups. Moreover, we show that these microparticles can evoke pro-survival and pro-angiogenic responses in primary human pulmonary endothelial cells.

MP-mediated transfer of membrane-associated receptors has been documented in a variety of cell types [26]. MPs may fully or partially fuse with the target cell, allowing for a complete or selective transfer of contents including membrane and cytosolic proteins, bioactive lipids, or even whole cell organelles [26–28]. The mechanism, depending on MP origin and cell type, is thought to involve integrin α4 or annexin V/phosphatidylserine signalling [26]. The role of microparticles in disease may be beneficial or detrimental, depending on the cellular source. For instance, MPs from animals with hypoxia-induced PH inhibit endothelium-dependent vasoreactivity of isolated pulmonary arteries and decrease nitric oxide production in isolated pulmonary endothelial cells [29]. On the other hand platelet or T-cell-derived MPs are thought to have a pro-proliferative and pro-angiogenic effects [30]. Our results support the notion that MPs secreted by endothelial cells in conditions of metabolic stress promote endothelial repair and counteract endothelial damage [31].

Pro-proliferative and pro-angiogenic effects of CTEPH EMPs were particularly evident in the presence of exogenous TGF-β. This may be explained, at least in part, by endoglin-mediated increase in TGF-β-induced smad1,5,8 phosphorylation. TGF-β regulates cell proliferation, differentiation, migration, synthesis of the extracellular matrix [32, 33] and promotes endothelial cell survival by activating ALK1/Smad 1/5/8 signalling [15, 34]. Interestingly, a weak stimulatory effect was also observed in the absence of exogenous TGF-β, likely to result from the effects induced by other components of EMPs. For instance, EMPs generated by apoptotic endothelial cells contain miR-126, known to promote endothelial angiogenesis [35, 36]. In addition, CTEPH EMP fraction contained plasminogen activator inhibitor 1 (Serpin E1) and traces of urokinase plasminogen activator (uPA). Serpine 1 is a serine protease inhibitor that stabilizes capillary vessel structure, regulates cell invasive behaviour and enhances TGF-β signaling [37, 38].

Reparative actions of CTEPH MPs may be attributed to endothelial endoglin^+^ MP fraction but the precise mechanism will require further studies. Confocal microscopy analysis documented the presence of microparticle-derived HA-tagged L-endoglin in recipient endothelial cells, suggesting a possibility of a functional receptor transfer. Recycling of membrane receptors carried by microparticles has been shown in various cell types [39–44]. Future studies will need to determine whether the recombinant endoglin was indeed recycled to the surface and remained functional. Of interest, platelet-derived MPs can increase surface expression of chemokine receptor CXCR4 by modification of receptor transfer, internalisation and externalisation as well as modified gene regulation [26, 45]. The type of endoglin microparticle carrier will also need further investigation. While microvesicles budding off the plasma membrane are most likely to play a role, exosomes can also carry surface proteins of their mother cells: exosomes from antigen presenting cells harbor MHCII on their surface, exosomes from reticulocytes contain the transferrin receptor, and exosomes from T-cells carry the TCR/CD3/zeta complex [46–48]. It is conceivable that local accumulation of endoglin^+^ microparticles in distal vasculature may promote angiogenesis and counteract the effects of Sol.Eng or other factors inducing endothelial senescence. Microparticle-mediated endoglin delivery may also have potential therapeutic implications in angiogenic disorders associated with abnormal endoglin function, such as hereditary hemorrhagic telangiectasia [49].

## Conclusions

Our study shows that CTEPH blood contains increased levels of endoglin^+^ endothelial microparticles and that these microparticles induce pro-survival and pro-angiogenic responses in human pulmonary endothelial cells, likely to reflect a healing mechanism set to counteract the effects of vascular occlusion and endothelial damage.

## Additional file

Additional file 1: Figure S1. Endothelial tube formation induced by CTEPH microparticles. HPAECs were cultured on Matrigel in serum-reduced (0.5 % serum) medium with different numbers of CTEPH microparticles, as indicated. Tube formation was scored after 18 h of incubation. ***P* < 0.01, comparison with untreated controls. **Figure S2.** Tube formation in HPAECs incubated with TGF-β and MPs. Representative images of tube formation in HPAECs incubated with MP fractions (full and endoglin^−^) from healthy and CTEPH plasma, with or without TGF-β (10 ng/mL;18 h). Bar = 50 μm. **Figure S3.** Endoglin levels in full MP fractions (containing endoglin^+^ MPs) and endoglin-depleted (endoglin^−^) MP fractions. Endoglin^+^ MPs were removed by Dynabeads-mediated immunoprecipitation and endoglin levels were measured with Human Endoglin/CD105 Quantikine ELISA Kit.****P* < 0.001, Student *t*-test, *n* = 6. **Figure S4.** Pro-angiogenic factors in CTEPH EMP fraction. (a) Proteome Profiler™ Human Angiogenesis Array membrane was incubated with CTEPH EMPs (image representative of *n* = 2). (R) shows reference spots, (1) corresponds to Serpin E1 and (2) corresponds to uPA, as indicated. Full information about the microarray layout can be found on https://resources.rndsystems.com/pdfs/datasheets/ary007.pdf. A schematic diagram of microarray is shown in (b). (DOCX 479 kb)

## Abbreviations

EMP: endothelial microparticles
CTEPH: chronic thromboembolic pulmonary hypertension
PE: pulmonary embolism
TGF-β: transforming growth factor β
FITC: Fluorescein isothiocyanate
VE-cadherin: vascular endothelial cadherin
