# Supplementary material for: Endothelium-derived microparticles from chronically thromboembolic pulmonary hypertensive patients facilitate endothelial angiogenesis
Source: J Biomed Sci. 2016 Jan 19;23:4. doi: 10.1186/s12929-016-0224-9 (PMC4717540; doi:10.1186/s12929-016-0224-9)
Supplement: Additional file 1: Figure S1. — Endothelial tube formation induced by CTEPH microparticles. HPAECs were cultured on Matrigel in serum-reduced (0.5 % serum) medium with different numbers of CTEPH microparticles, as indicated. Tube formation was scored after 18 h of incubation. **P < 0.01, comparison with untreated controls. Figure S2. Tube formation in HPAECs incubated with TGF-β and MPs. Representative images of tube formation in HPAECs incubated with MP fractions (full and endoglin−) from healthy and CTEPH plasma, with or without TGF-β (10 ng/mL;18 h). Bar = 50 μm. Figure S3. Endoglin levels in full MP fractions (containing endoglin+ MPs) and endoglin-depleted (endoglin−) MP fractions. Endoglin+ MPs were removed by Dynabeads-mediated immunoprecipitation and endoglin levels were measured with Human Endoglin/CD105 Quantikine ELISA Kit.***P < 0.001, Student t-test, n = 6. Figure S4. Pro-angiogenic factors in CTEPH EMP fraction. (a) Proteome Profiler™ Human Angiogenesis Array membrane was incubated with CTEPH EMPs (image representative of n = 2). (R) shows reference spots, (1) corresponds to Serpin E1 and (2) corresponds to uPA, as indicated. Full information about the microarray layout can be found on https://resources.rndsystems.com/pdfs/datasheets/ary007.pdf. A schematic diagram of microarray is shown in (b). (DOCX 479 kb) [file 12929_2016_224_MOESM1_ESM.docx]

**Additional File 1**

**“Endothelium-derived microparticles from chronically thromboembolic pulmonary hypertensive patients facilitate endothelial angiogenesis” Belik et al.**

**
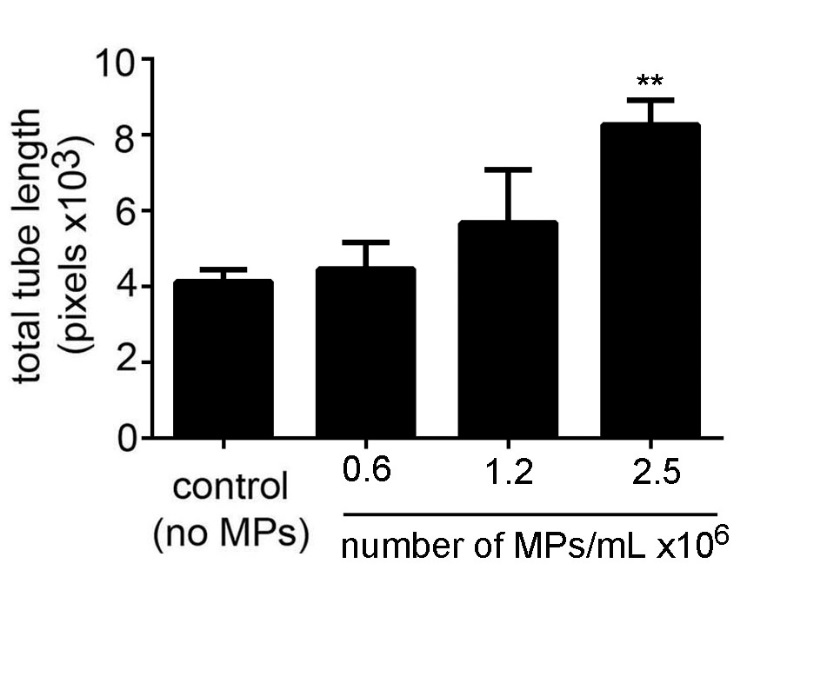
**

**Figure S1 Endothelial tube formation induced by CTEPH microparticles.** HPAECs were cultured on Matrigel in serum-reduced (0.5% serum) medium with different numbers of CTEPH microparticles, as indicated. Tube formation was scored after 18 hours of incubation. **P<0.01, comparison with untreated controls.

**
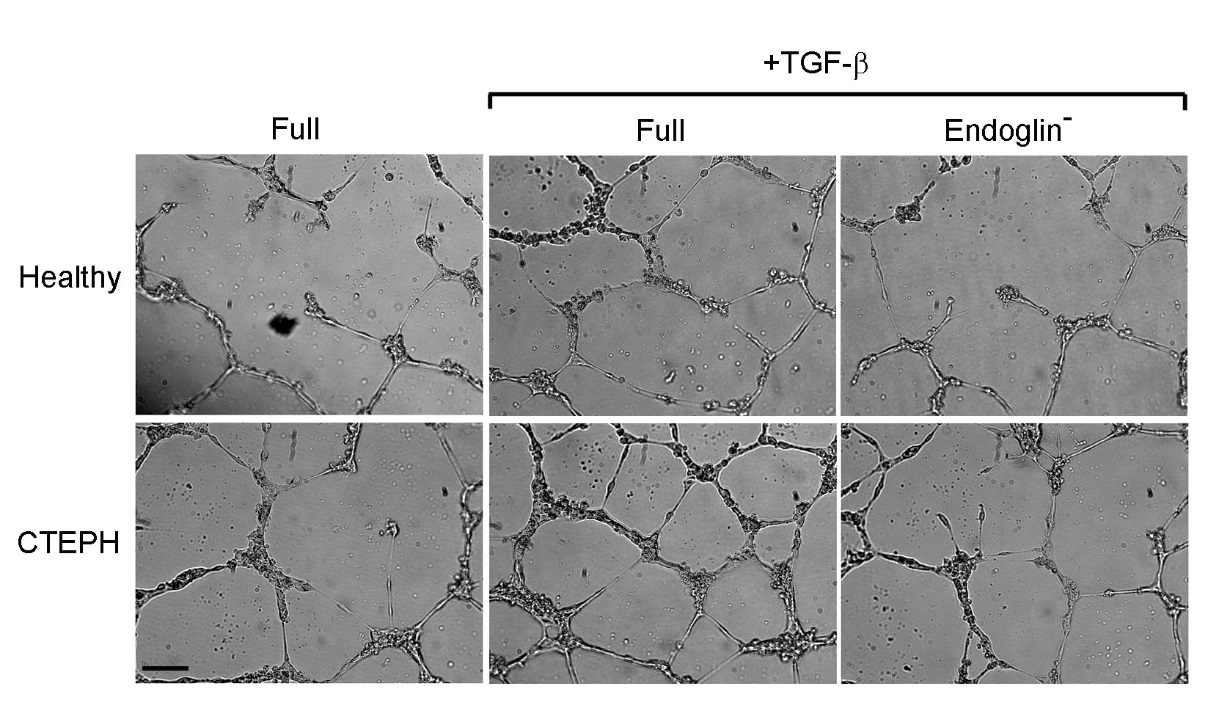
**

**Figure S2.** Tube formation in HPAECs incubated with TGF-β and MPs. Representative images of tube formation in HPAECs incubated with MP fractions (full and endoglin^-^) from healthy and CTEPH plasma, with or without TGF-β (10ng/mL;18 hours). Bar=50 µm

**
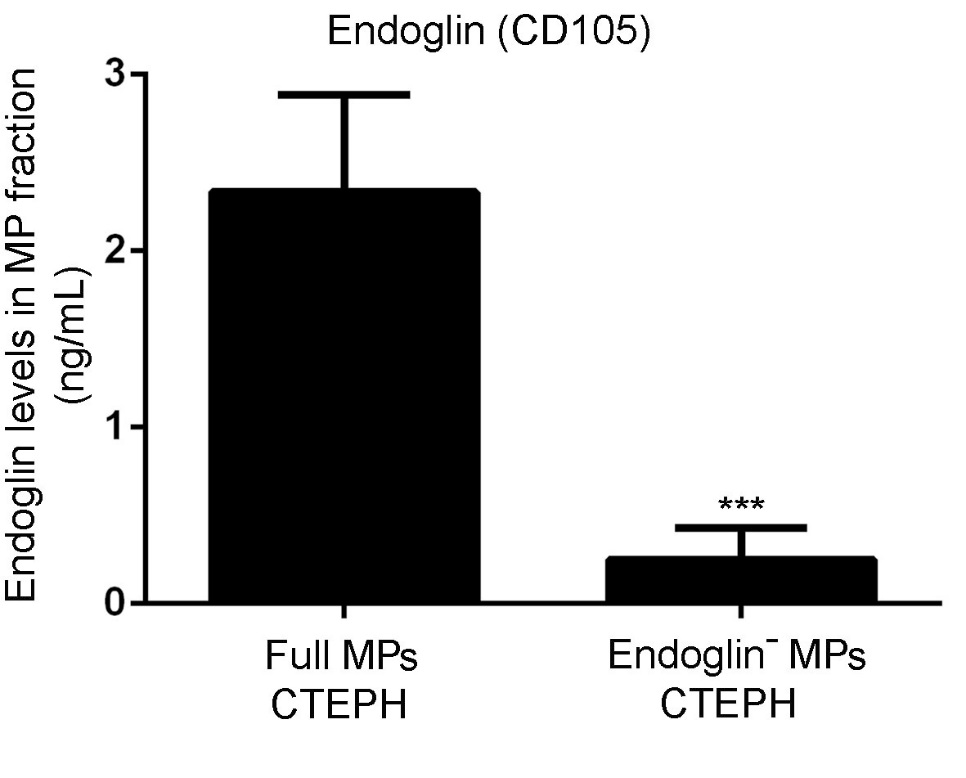
**

**Figure S3. Endoglin levels in full MP fractions (containing endoglin^+^ MPs) and endoglin-depleted (endoglin^-^) MP fractions**. Endoglin^+^ MPs were removed by Dynabeads-mediated immunoprecipitation and endoglin levels were measured with Human Endoglin/CD105 Quantikine ELISA Kit.***P<0.001, Student t-test, n=6.

**
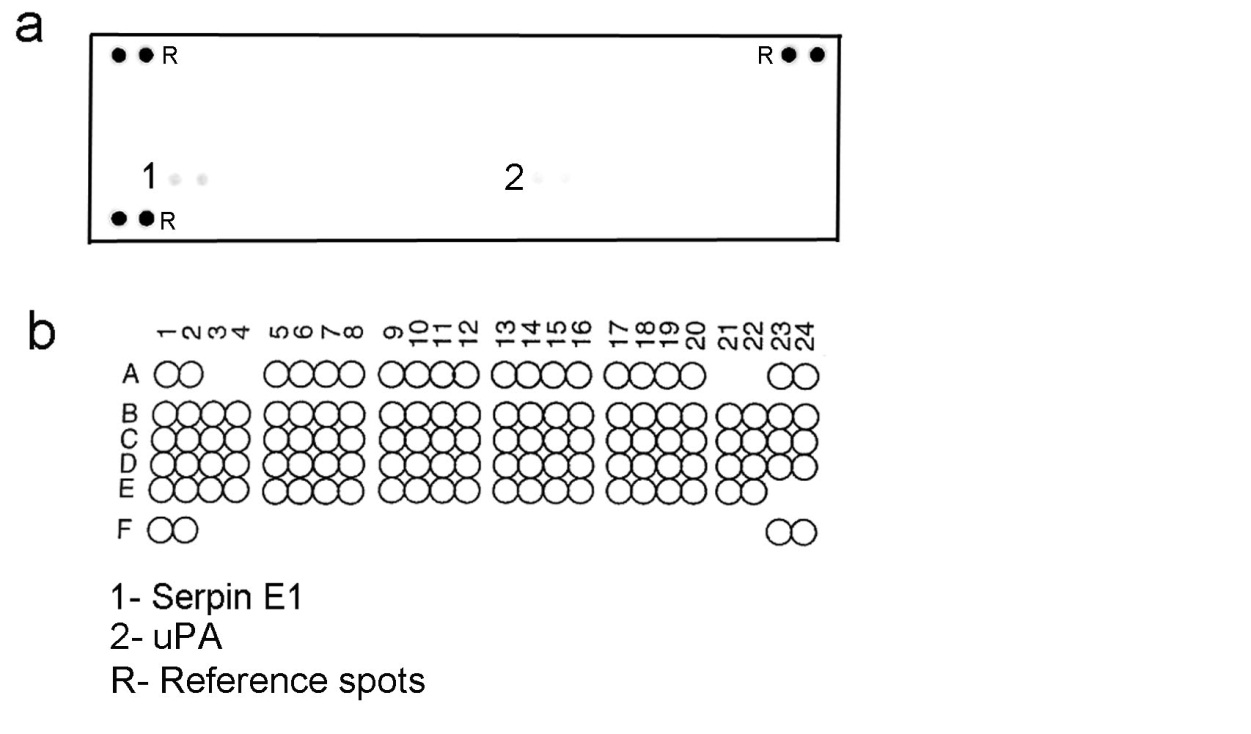
**

**Figure S4. Pro-angiogenic factors in CTEPH EMP fraction.** (a) Proteome Profiler™ Human Angiogenesis Array membrane was incubated with CTEPH EMPs (image representative of n=2). (R) shows reference spots, (1) corresponds to Serpin E1 and (2) corresponds to uPA, as indicated. Full information about the microarray layout can be found on https://resources.rndsystems.com/pdfs/datasheets/ary007.pdf. A schematic diagram of microarray is shown in (b).
